# Supplementary material for: Tensile straining of iridium sites in manganese oxides for proton-exchange membrane water electrolysers
Source: Nat Commun. 2024 Jan 2;15:95. doi: 10.1038/s41467-023-44483-6 (PMC10762142; doi:10.1038/s41467-023-44483-6)
Supplement: Supplementary file 1 — Supplementary Information [file 41467_2023_44483_MOESM1_ESM.pdf]

Supplementary Information for

Tensile straining of iridium sites in manganese oxides for proton-exchange membrane water electrolysers

Hui Su,[1,5,*] Chenyu Yang,[2,5] Meihuan Liu,[3] Xu Zhang,[4] Wanlin Zhou,[2] Yuhao Zhang,[2] Kun Zheng,[4] Shixun Lian,[1,*] and Qinghua Liu[2,*]

[1]Key Laboratory of Light Energy Conversion Materials of Hunan Province College, College of Chemistry and Chemical Engineering, Hunan Normal University, Changsha 410081, Hunan, China
[2]National Synchrotron Radiation Laboratory, University of Science and Technology of China, Hefei 230029, Anhui, China
[3] State Key Laboratory for Powder Metallurgy, Central South University, Changsha 410083, Hunan, China
[4]Beijing Key Laboratory of Microstructure and Properties of Solids, Faculty of Materials and Manufacturing, Beijing University of Technology, Beijing, 100124, China
[5] These authors contributed equally: Hui Su, Chenyu Yang.

*E-mail: suhui@hunnu.edu.cn; sxlian@hunnu.edu.cn; qhliu@ustc.edu.cn

Contents:

Supplementary Figs. 1-32;

Supplementary Tables 1-7;

Supplementary references 1-20.

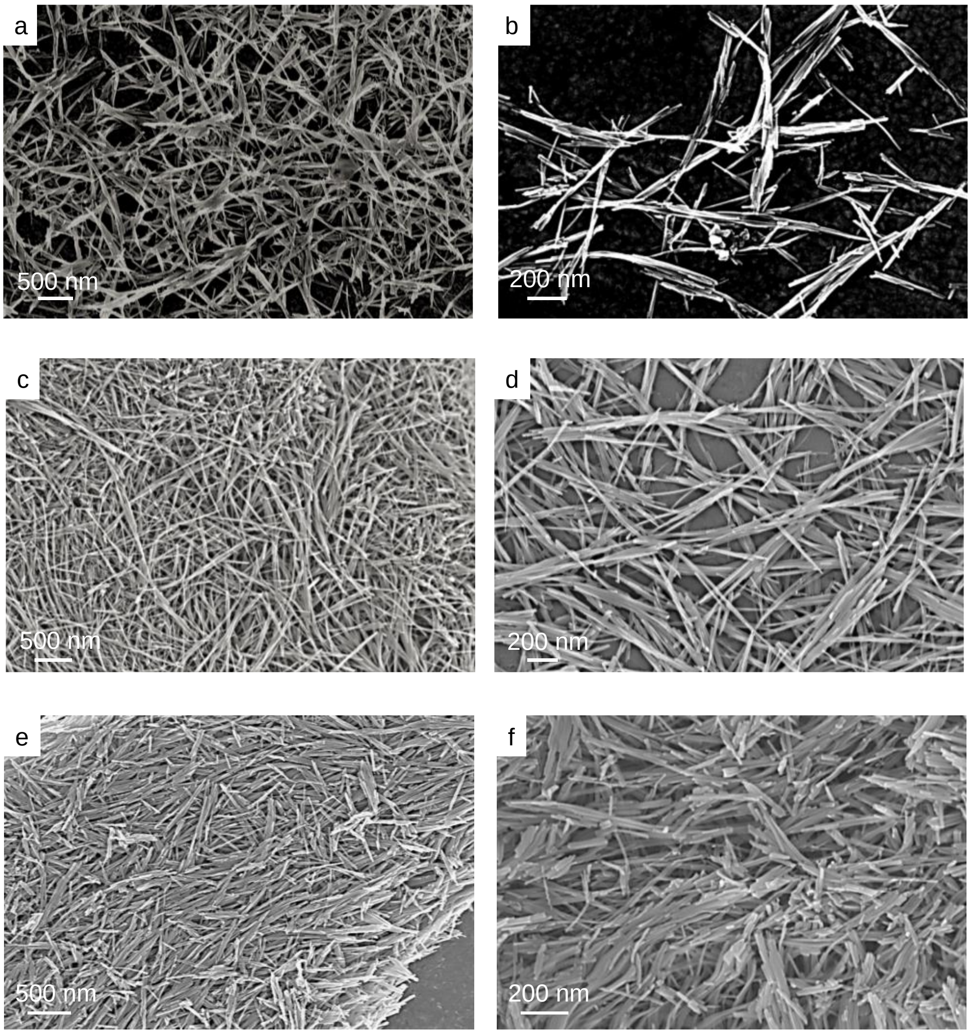

Supplementary Fig. 1 SEM images of (a) and (b) MnO_2, (c) and (d) Ir-MnO_2, (e) and (f) TS-Ir/MnO_2.

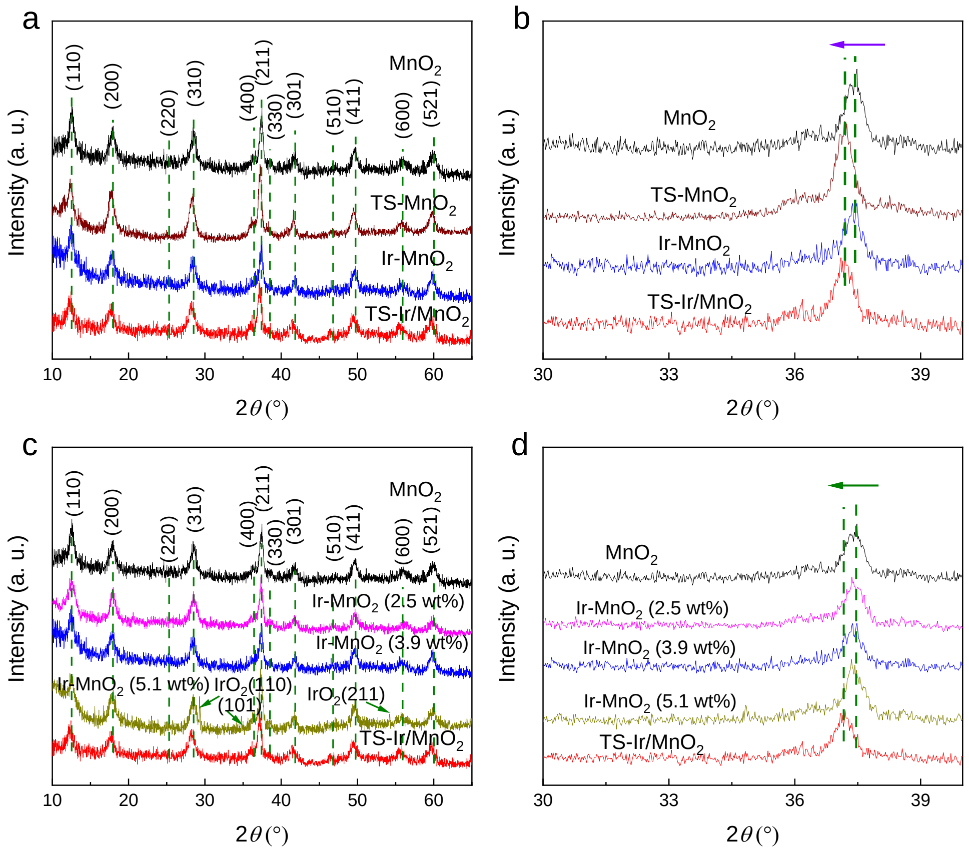

Supplementary Fig. 2 (a) and (b) XRD patterns for TS-Ir/MnO_2, Ir-MnO_2 and MnO_2 electrocatalysts. (c) and (d) XRD patterns for MnO_2, Ir-MnO_2 (2.5 wt%, 3.9 wt% and 5.1 wt%) and TS-Ir/MnO_2 electrocatalysts.

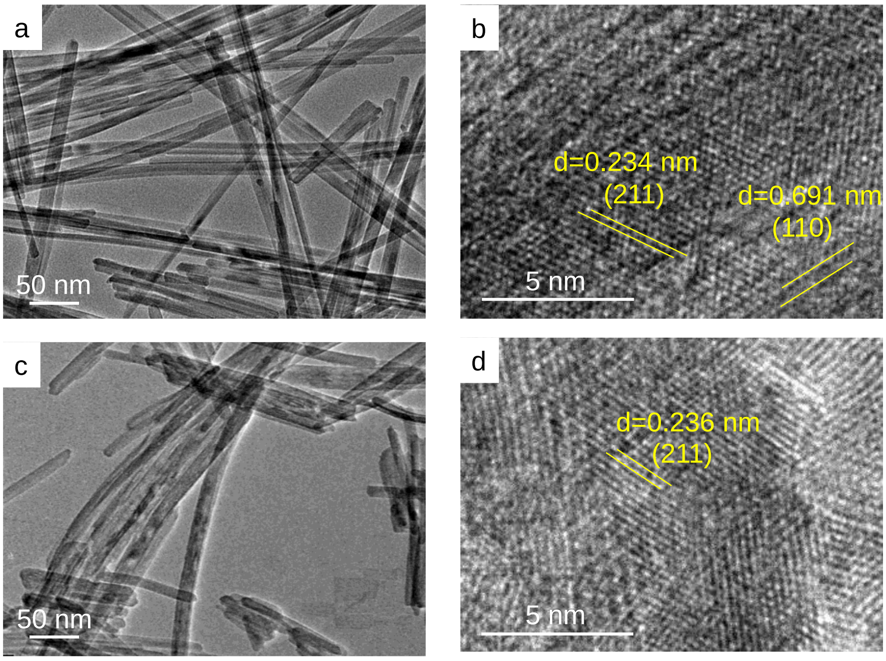

Supplementary Fig. 3 (a) TEM and (b) HRTEM images of MnO_2. (c) TEM and (b) HRTEM images of Ir-MnO_2.

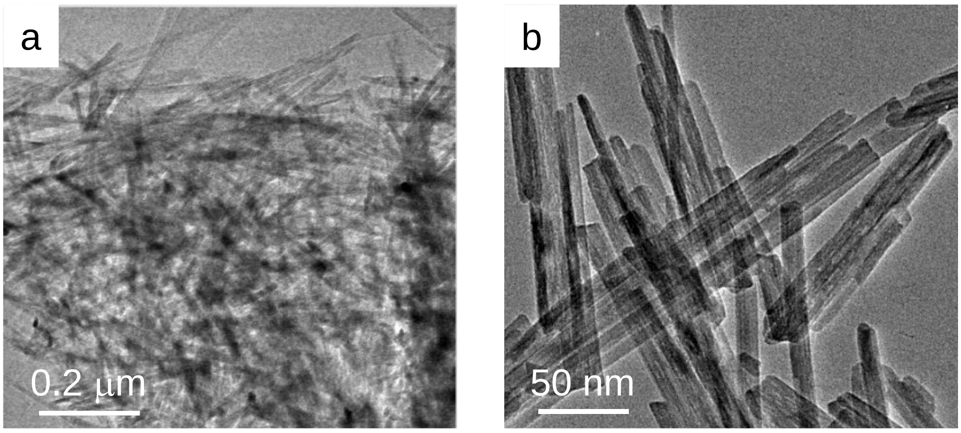

Supplementary Fig. 4 (a) and (b) TEM images of TS-Ir/MnO_2.

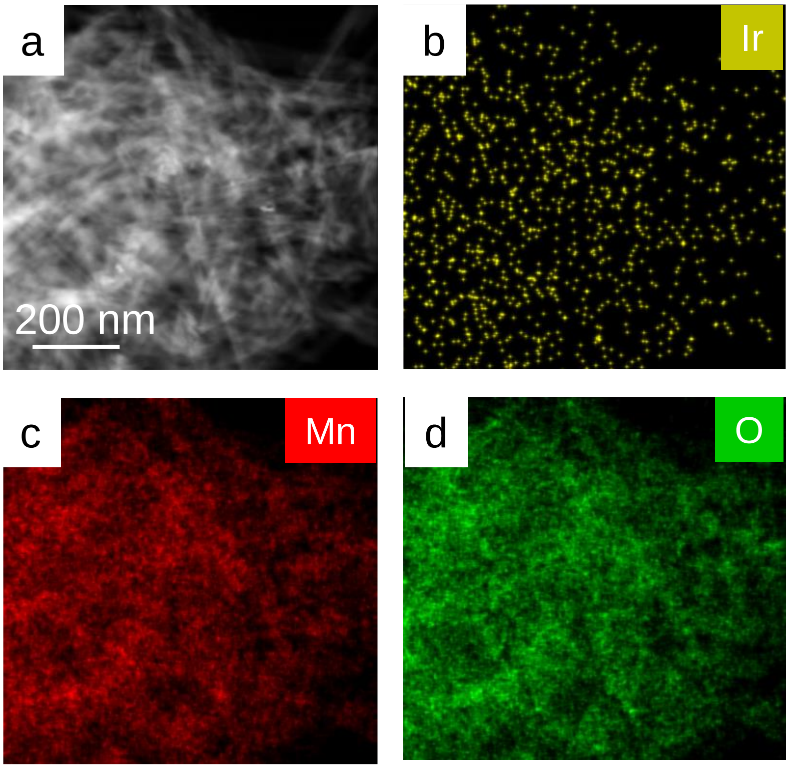

Supplementary Fig. 5 STEM-EDS mapping images for TS-Ir/MnO_2.

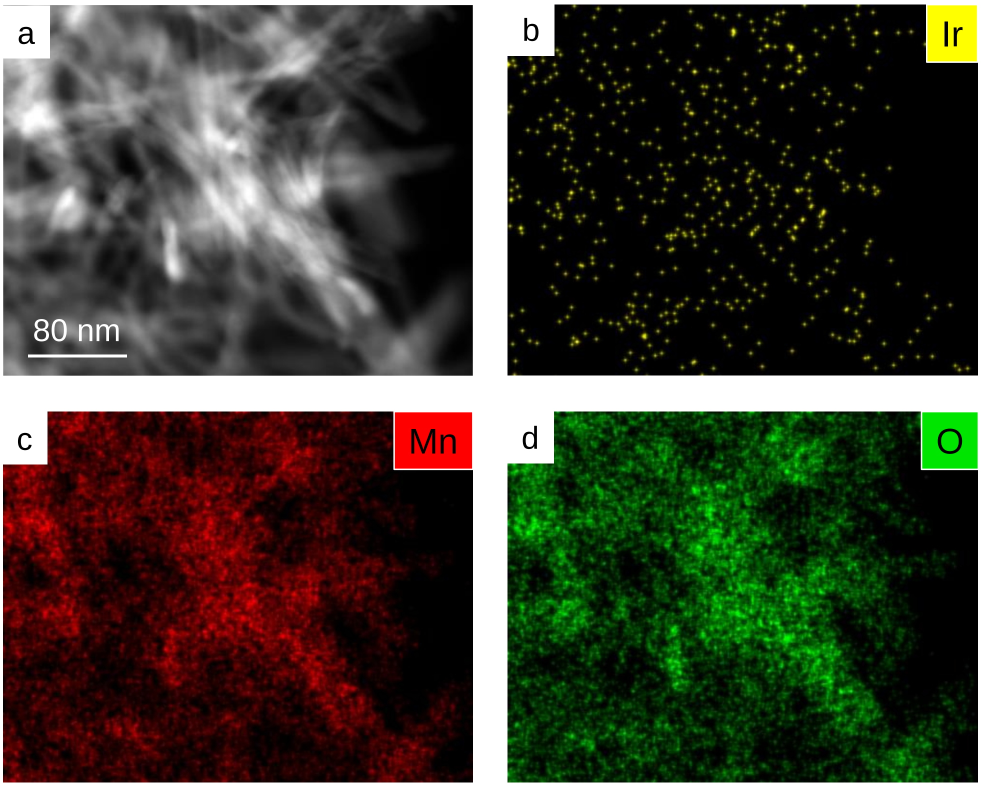

Supplementary Fig. 6 STEM-EDS mapping images for Ir-MnO_2.

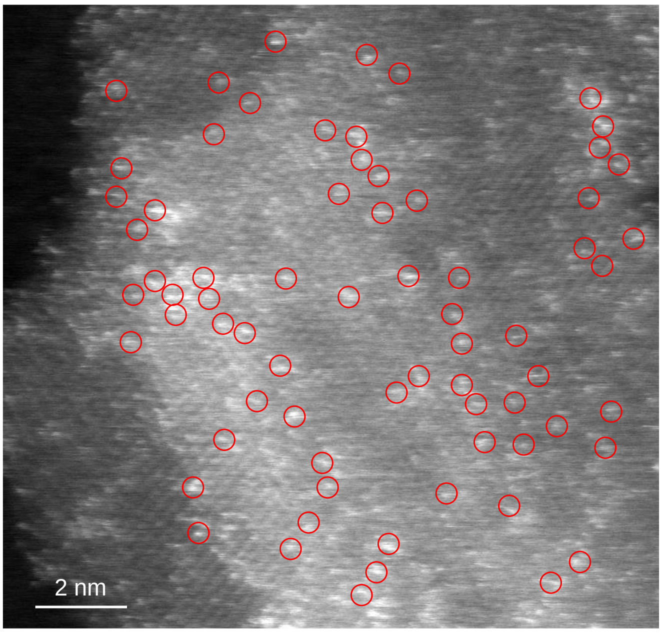

Supplementary Fig. 7 HAADF-TEM image for Ir-MnO_2 and inset red circles represent Ir sites.

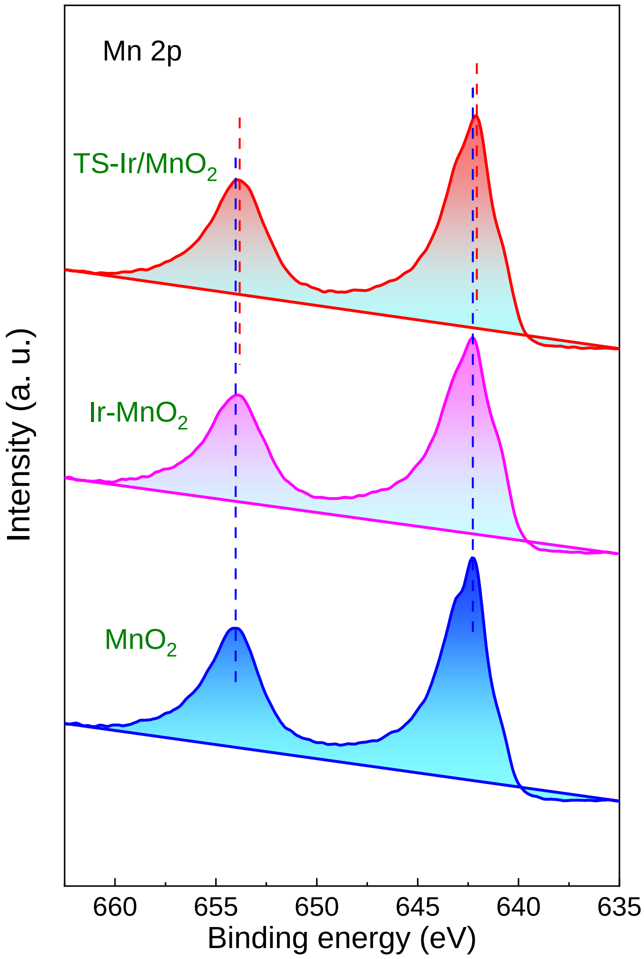

Supplementary Fig. 8 Mn 2p XPS spectra for MnO_2, Ir-MnO_2 and TS-Ir/MnO_2.

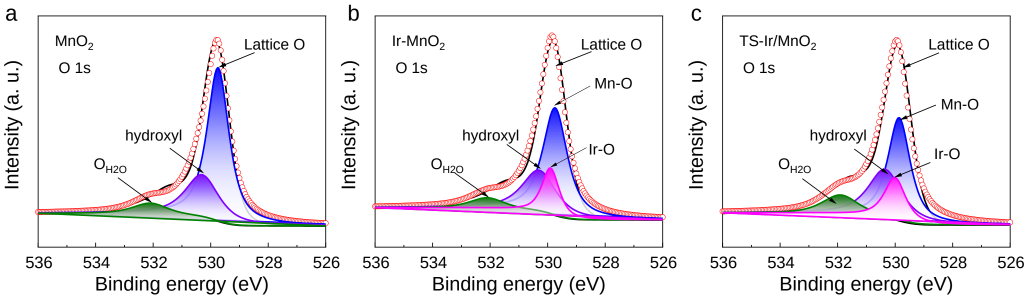

Supplementary Fig. 9 High-resolution spectra of O 1s for MnO_2 (a), Ir-MnO_2 (b) and TS-Ir/MnO_2 (c).

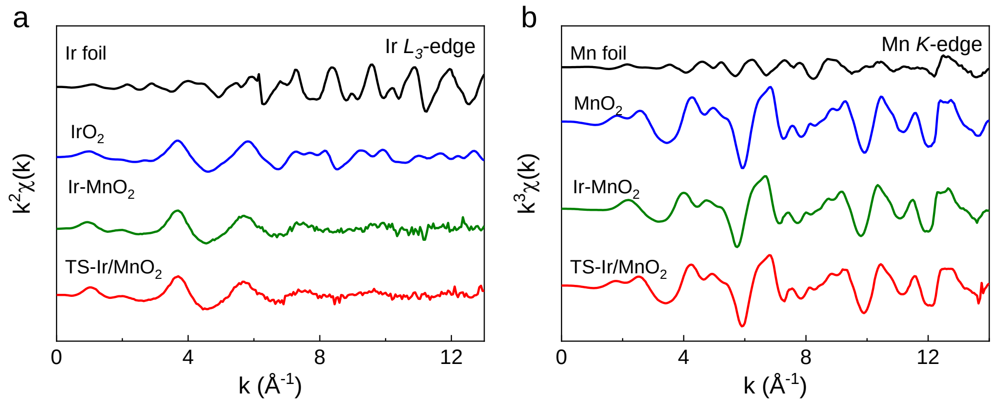

Supplementary Fig. 10 (a) $k^2\chi(k)$ curves of Ir L_3-edge EXAFS oscillation functions for TS-Ir/MnO_2, Ir-MnO_2, IrO_2 and Ir foil. (b) $k^3\chi(k)$ curves of Mn *K*-edge EXAFS oscillation functions for TS-Ir/MnO_2, Ir-MnO_2, MnO_2 and Mn foil.

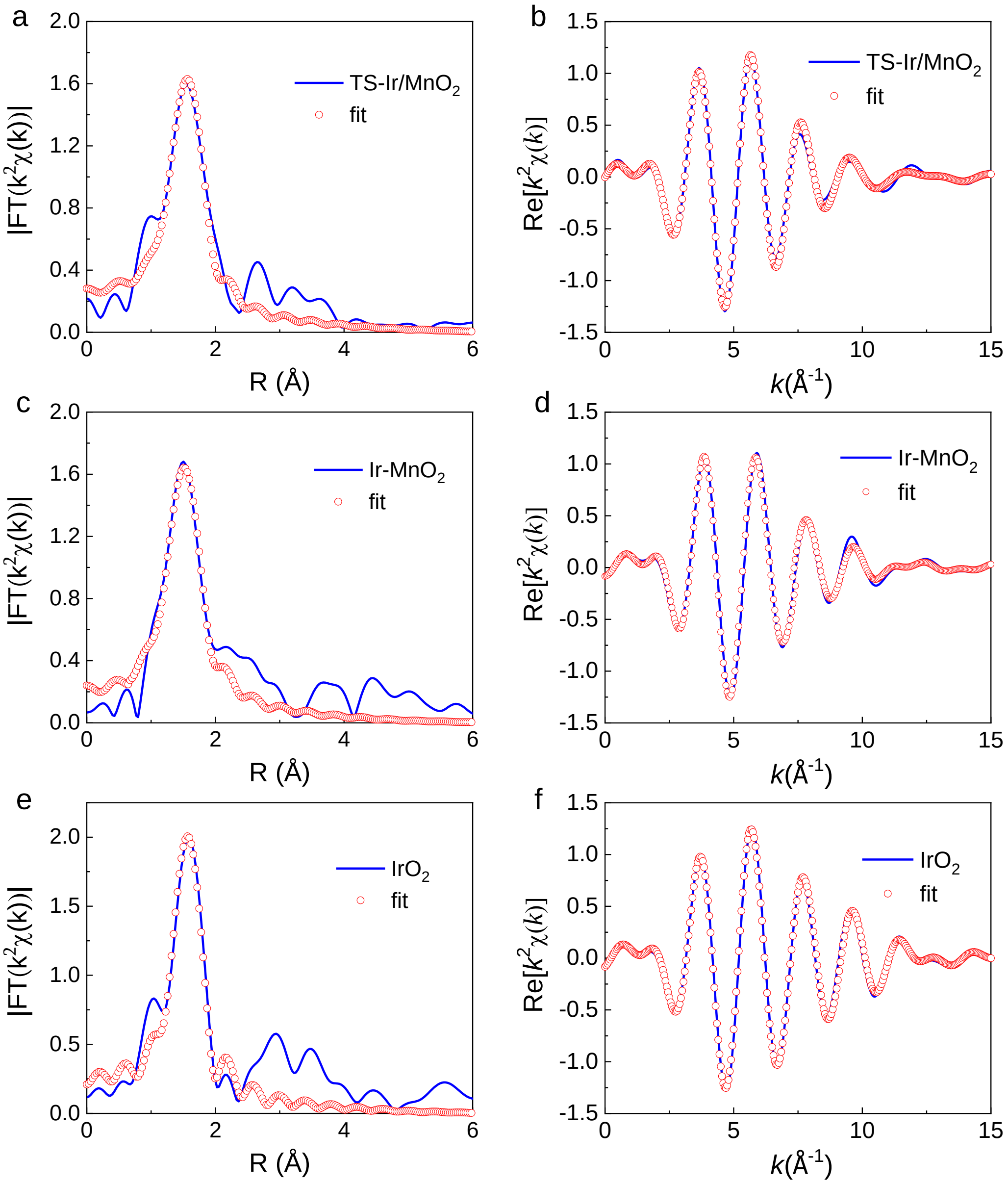

Supplementary Fig. 11 The fitting curves of k^2-weighted Ir L_3-edge EXAFS spectra and corresponding k^2-weighted FT of Ir L_3-edge EXAFS oscillation functions for (a, b) TS-Ir/MnO_2, (c, d) Ir-MnO_2 and (e, f) IrO_2.

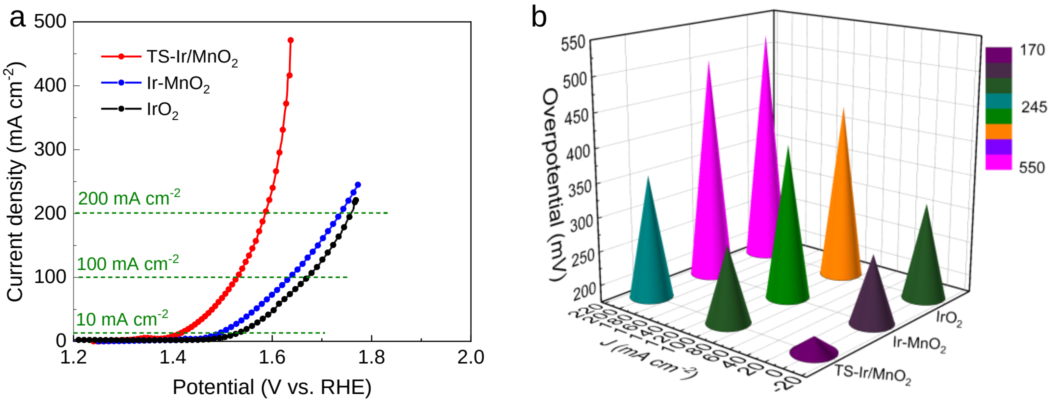

Supplementary Fig. 12 (a) OER polarization curves of TS-Ir/MnO_2, Ir-MnO_2, IrO_2. (b) The corresponding overpotentials at current densities of 10, 100 and 200 mA cm^{-2}.

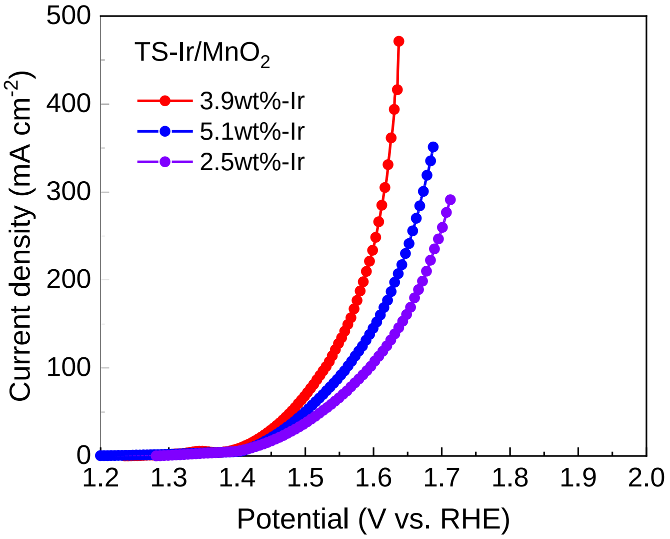

Supplementary Fig. 13 OER polarization curves of TS-Ir/MnO_2 with different metal loading of Ir.

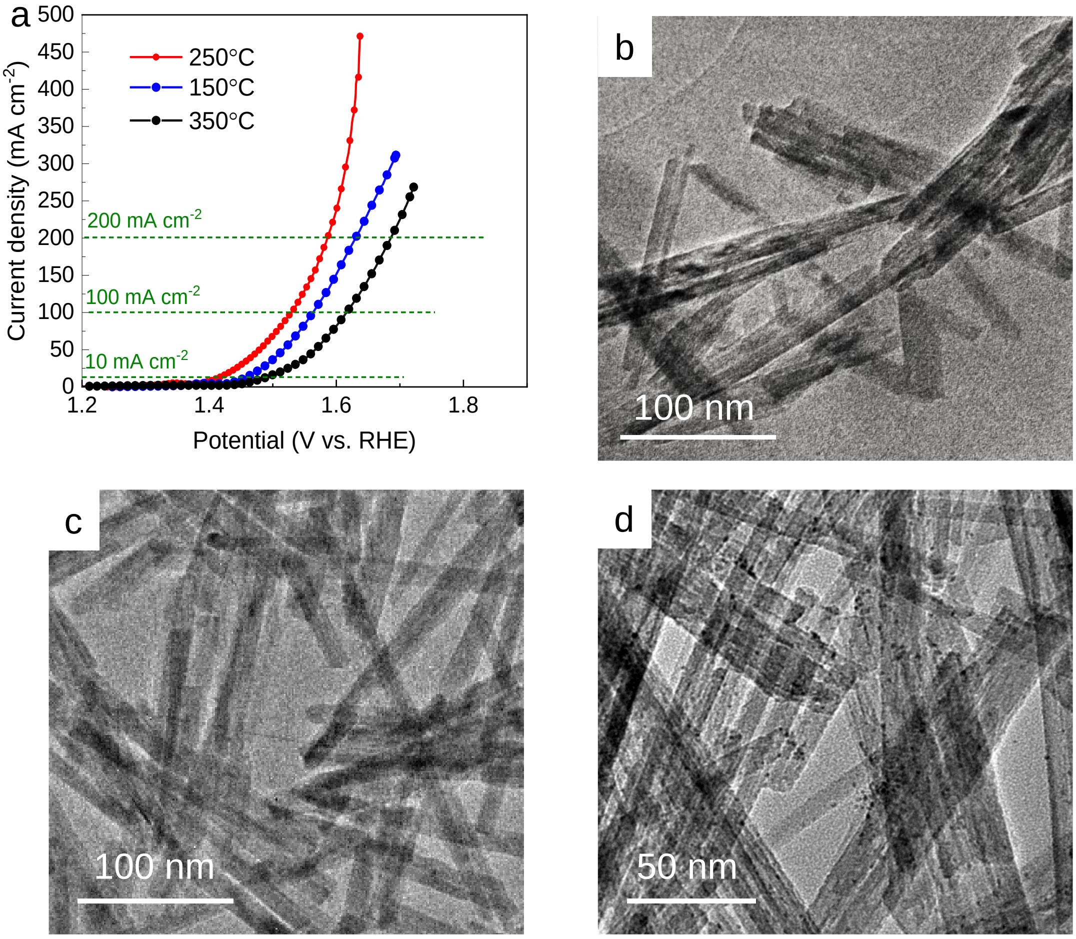

Supplementary Fig. 14. (a) Linear sweep voltammetry (LSV) curves for Ir electrocatalysts thermal annealed at 150, 250 and 350 ˚C. TEM images of Ir-based electrocatalysts annealed at (b) 250 ˚C, (c) 150 ˚C and (d) 350 ˚C.

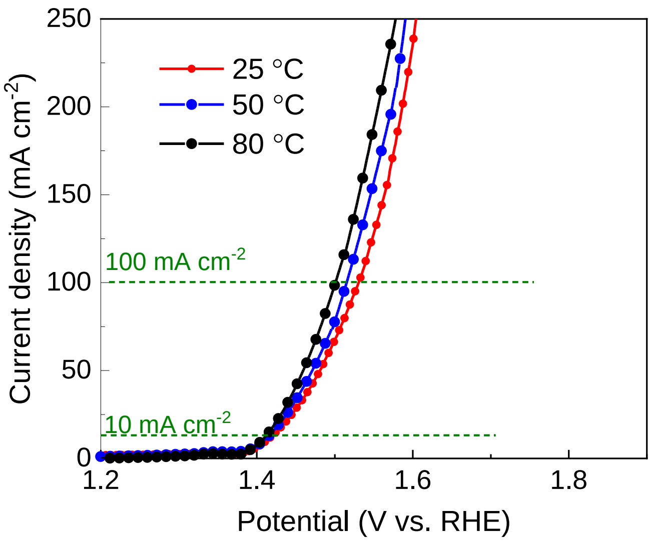

Supplementary Fig. 15. Linear sweep voltammetry (LSV) curves of TS-Ir/MnO_2 under 25, 50 and 80 ˚C in 0.1 M $HClO_4$.

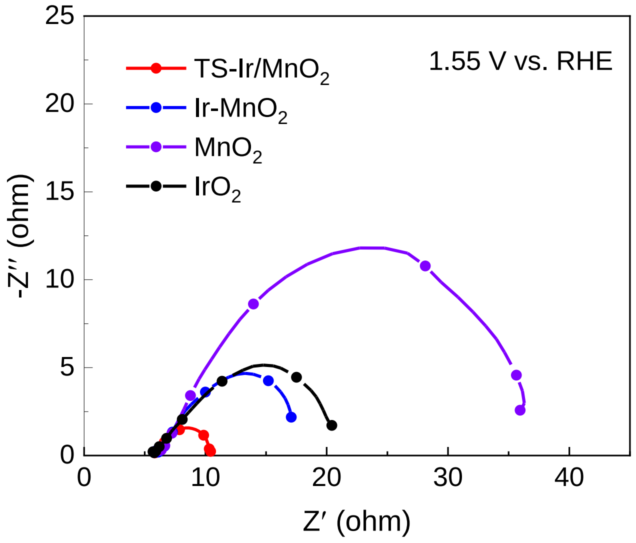

Supplementary Fig. 16. Nyquist plots measured at 1.55 V (*vs*. RHE) of TS-Ir/MnO_2, Ir-MnO_2, MnO_2 and IrO_2.

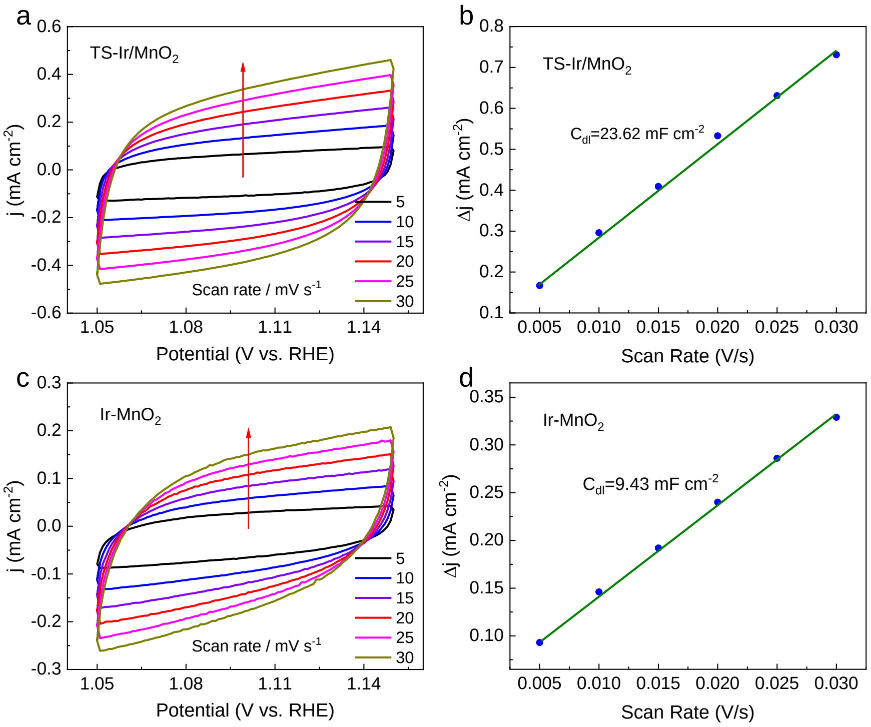

Supplementary Fig. 17 Double-layer capacitance measurements. (a), (c) CVs were conducted in a non-Faradaic region of voltammogram at the following scan rate: 5, 10, 15, 20, 25 and 30 mV s^{-1} for TS-Ir/MnO_2 and Ir-MnO_2, respectively. (b), (d) The difference in charging currents variation at an underpotential plotted against scan rate for estimation of double-layer capacitance (C_{dl}) for TS-Ir/MnO_2 and Ir-MnO_2, respectively.

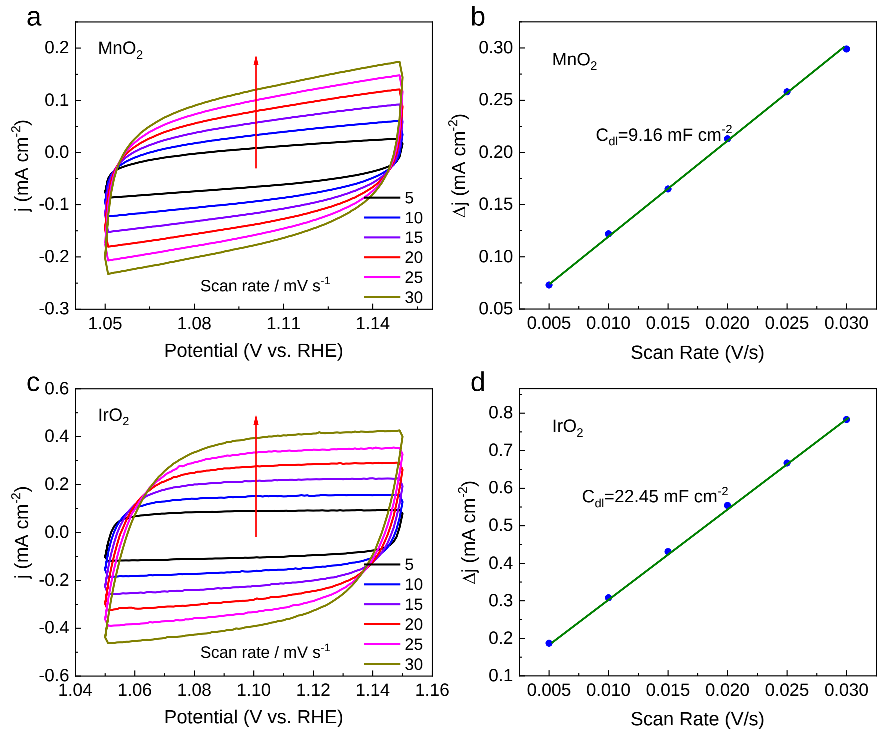

Supplementary Fig. 18 Double-layer capacitance measurements. (a), (c) CVs were conducted in a non-Faradaic region of voltammogram at the following scan rate: 5, 10, 15, 20, 25 and 30 mV s^{-1} for MnO_2 and IrO_2, respectively. (b), (d) The difference in charging currents variation at an underpotential plotted against scan rate for estimation of double-layer capacitance (C_{dl}) for MnO_2 and IrO_2, respectively.

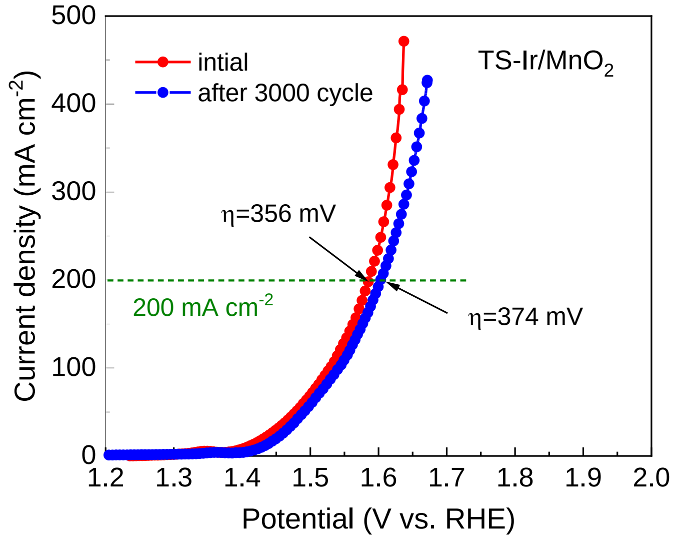

Supplementary Fig. 19 OER polarization curves of TS-Ir/MnO_2 electrocatalyst before and after 3000 electrochemical test cycles.

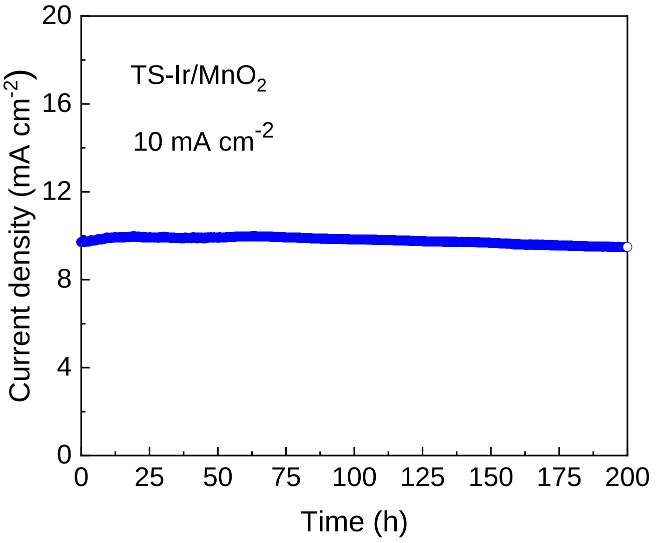

Supplementary Fig. 20 Chronoamperometry measurement of TS-Ir/MnO_2.

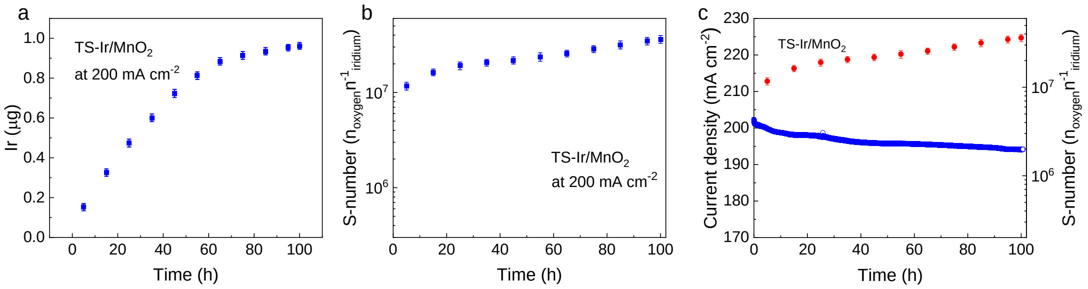

Supplementary Fig. 21 (a) Dissolved content of metal Ir, (b) the calculated constant S-number under 200 mA cm^{-2}. (c) Chronoamperometry measurement and S-number for TS-Ir/MnO_2.

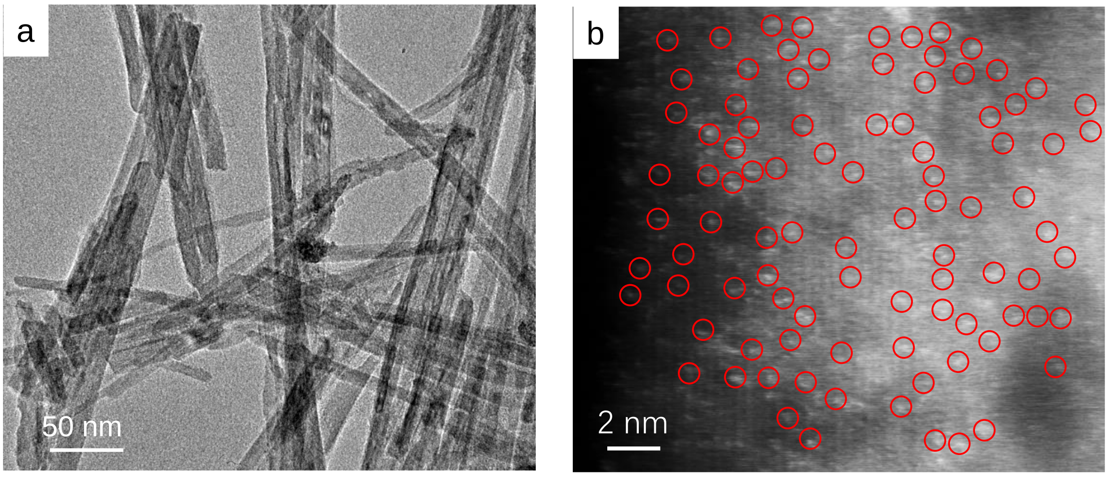

Supplementary Fig. 22 (a) TEM and (b) HAADF-TEM images for TS-Ir/MnO_2 after electrochemical measurements.

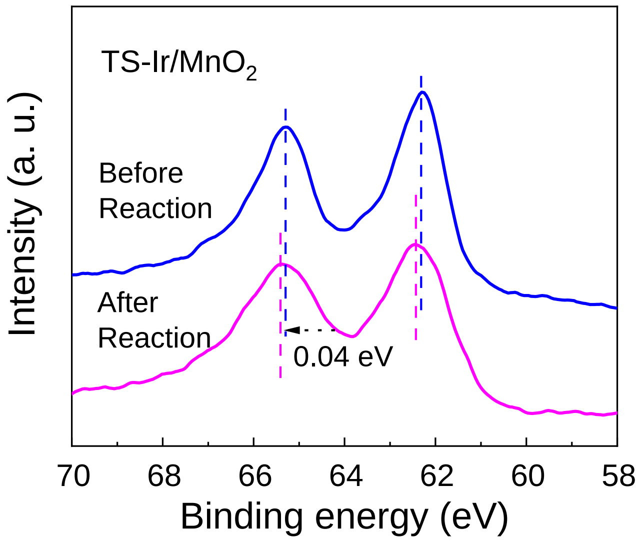

Supplementary Fig. 23 Ir 4f XPS spectra for TS-Ir/MnO_2 before and after electrochemical measurements.

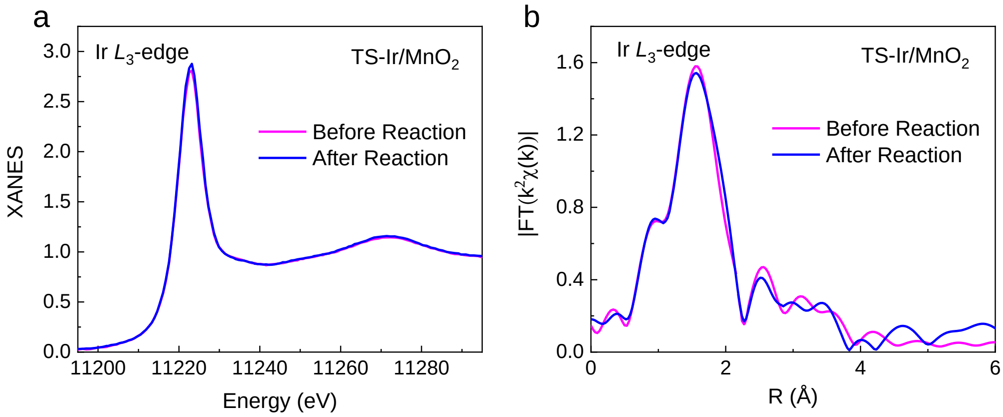

Supplementary Fig. 24 (a) Ir L_3-edge XANES spectra and (b) Fourier transforms (FTs) of the Ir L_3-edge EXAFS oscillations of TS-Ir/MnO_2 before and after electrochemical measurements.

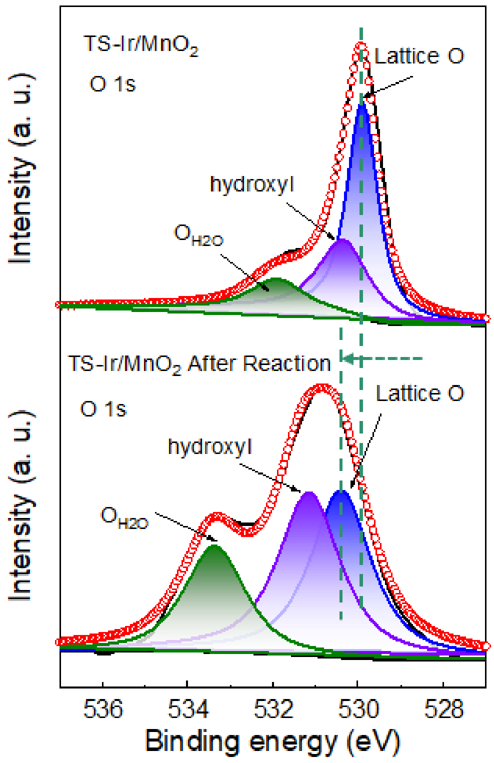

Supplementary Fig. 25 O 1s XPS spectra for TS-Ir/MnO_2 before and after electrochemical measurements.

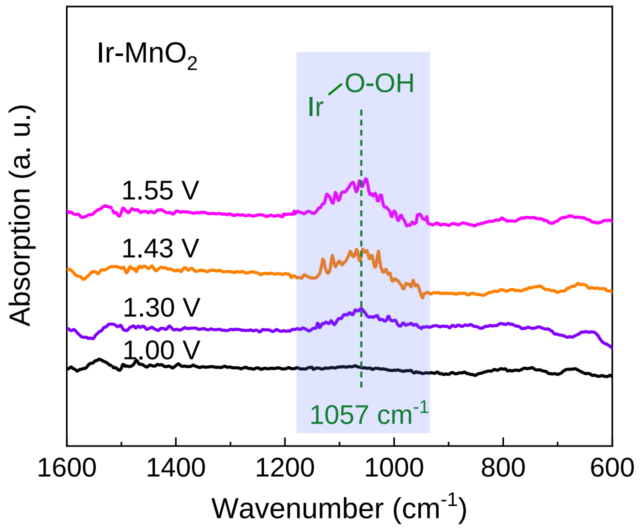

Supplementary Fig. 26 In situ SRIR spectroscopy measurements of Ir-MnO_2 at various potentials.

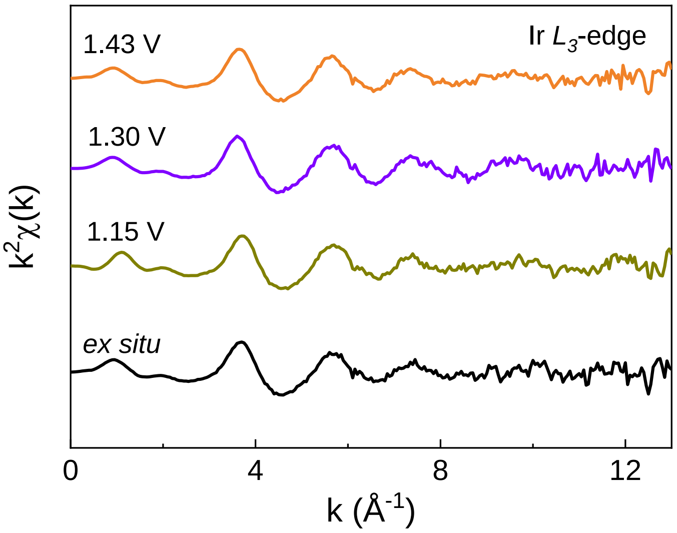

Supplementary Fig. 27 $k^2\chi(k)$ curves of Ir L_3-edge EXAFS oscillation functions for TS-Ir/MnO_2 under different applied potentials.

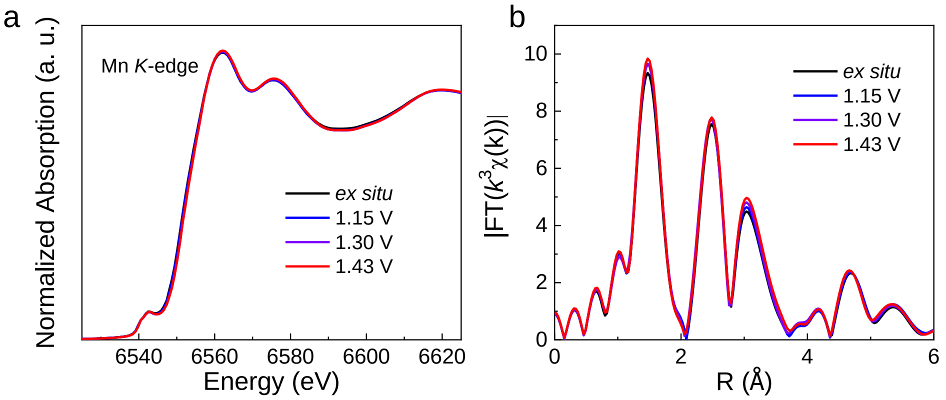

Supplementary Fig. 28 (a) Mn K-edge XANES spectra and (b) Fourier transforms (FTs) of the Mn K-edge EXAFS oscillations of TS-Ir/MnO_2 under different applied potentials.

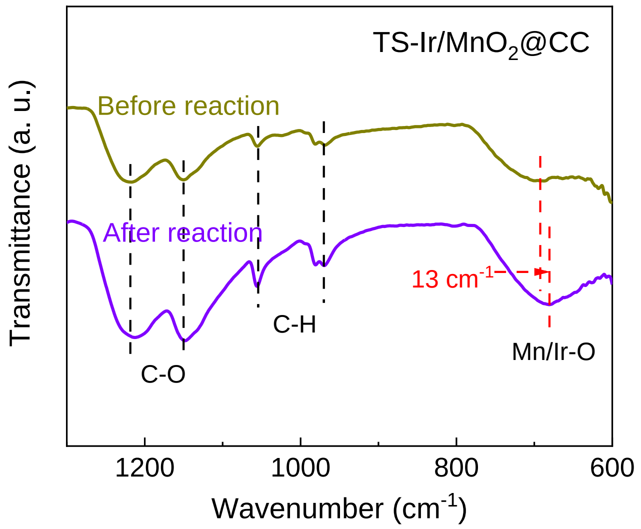

Supplementary Fig. 29 The FTIR image of TS-Ir/MnO_2@CC before and after isotopically labelled OER measurements.

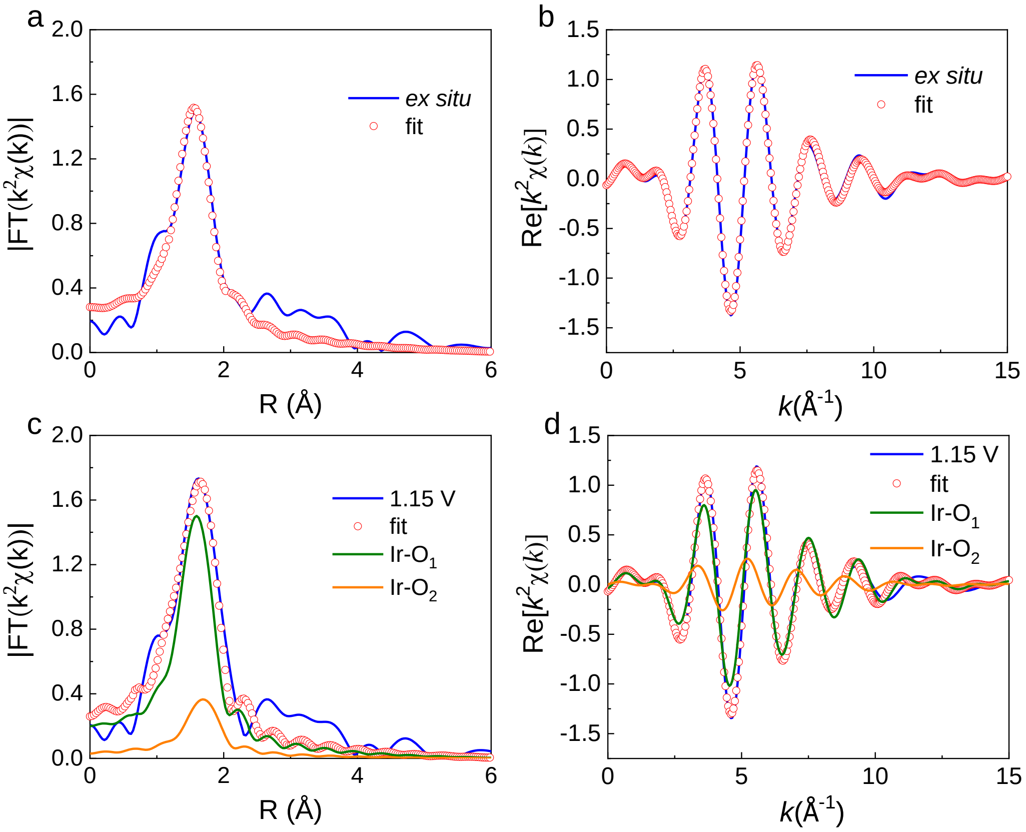

Supplementary Fig. 30 In situ XAFS measurements for TS-Ir/MnO_2. The fitting curves of k^2-weighted EXAFS spectra and the Re($k^2\chi(k)$) oscillation curves under ex situ conditions (a and b) and 1.15 V conditions (c and d).

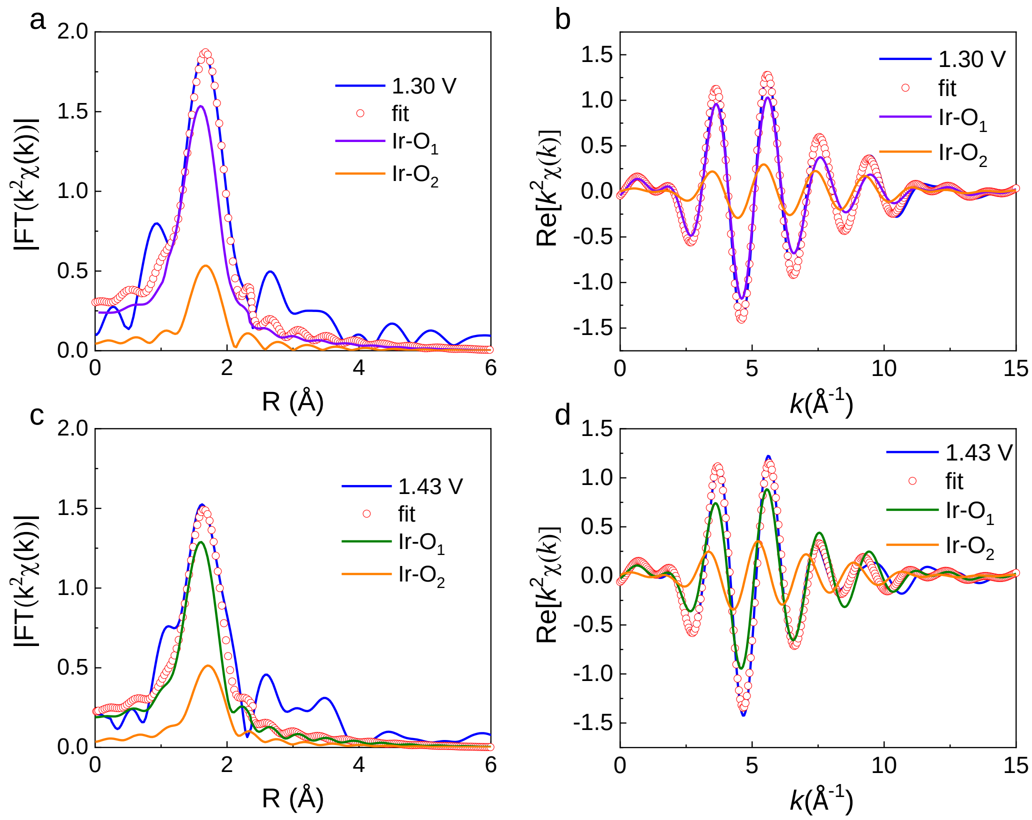

Supplementary Fig. 31 In situ XAFS measurements for TS-Ir/MnO_2. The fitting curves of k^2-weighted EXAFS spectra and the Re($k^2\chi$(k)) oscillation curves under 1.30 V conditions (a and b) and 1.43 V conditions (c and d).

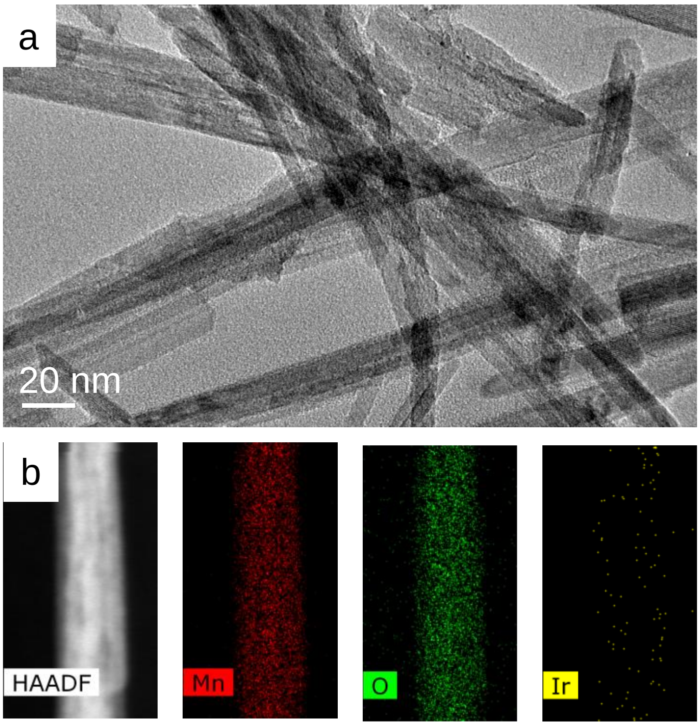

Supplementary Fig. 32 (a) TEM image and (b) STEM-EDS mapping images for TS-Ir/MnO_2 after the PEM electrolyser device test.

Supplementary Table 1. The apparent bond length of Ir–O/Mn–O in all samples.

Sample	Apparent bond length (Ir–O)	Apparent bond length (Mn–O)
TS-Ir/MnO_2	1.54 Å	1.50 Å
Ir-MnO_2	1.48 Å	1.47 Å
MnO_2	--	1.47 Å
IrO_2	1.60 Å	--

Supplementary Table 2 Structural parameters for TS-Ir/MnO_2, Ir-MnO_2 and IrO_2 electrocatalysts extracted from quantitative EXAFS curve-fitting using the ARTEMIS module of IFEFFIT.

Sample	Path	N	R (Å)	σ^2(10^{-3}Å^2)	ΔE_0(eV)	R-factor
TS-Ir/MnO_2	Ir-O	4.2±0.3	1.94±0.005	3.6±0.5	9.7±1.3	0.005
Ir-MnO_2	Ir-O	4.1±0.2	1.89±0.01	5.1±1.1	9.8±2.1	0.005
IrO_2	Ir-O	6	2.01±0.01	3.3±0.5	9.7±1.6	0.003

N, coordination number; R, bond length; σ^2 Debye-Waller factor; ΔE_0 inner potential shift.

Supplementary Table 3. Comparison of OER activity of TS-Ir/MnO_2 electrocatalyst with other recently reported catalysts in acid solution according to catalytic kinetic parameter (Tafel slope).[1-11]

Catalyst	Electrolyte	Support	Tafel slope (mV dec^{-1})	Ref.
TS-Ir/MnO_2	0.1 M $HClO_4$	Carbon cloth	56.6	This work
Ir-MnO_2	0.1 M $HClO_4$	Carbon cloth	101.2	This work
IrMnOF@Ir	0.1 M $HClO_4$	Carbon paper	58.3	[1]
Ir/δ-MnO_2	0.5 M H_2SO_4	Carbon Paper	123	[2]
$(Mn_{0.8}Ir_{0.2})O_2$:10F	0.5 M H_2SO_4	Ti-foils	38	[3]
IrO_x/Zr_2ON_2	0.5 M H_2SO_4	RDE	48	[4]
GB-$Ta_{0.1}Tm_{0.1}Ir_{0.8}O_{2-δ}$	0.5 M H_2SO_4	RDE (Au)	64	[5]
IrO_2/GCN	0.5 M H_2SO_4	RDE	57	[6]
12Ru/MnO_2	0.1 M $HClO_4$	GCE	29.4	[7]
H/d-MnO_x/RuO_2	0.5 M H_2SO_4	GCE	43.8	[8]
$Li_{0.52}RuO_2$	0.5 M H_2SO_4	GCE	83.3	[9]
90-Co-MnO_2	0.1 M $HClO_4$	GCE	158	[10]
IrO_2/a-MnO_2	0.1 M $HClO_4$	GCE	74	[11]

Note: RDE, rotating disk electrode; GCE, glassy carbon electrode

Supplementary Table 4. The calculated C_{dl}, ECSA and R_F for TS-Ir/MnO_2, Ir-MnO_2, MnO_2 and IrO_2.

	TS-Ir/MnO_2	Ir-MnO_2	MnO_2	IrO_2
C_{dl} (mF cm^{-2})	23.62	9.43	9.16	22.45
ECSA	674.86	269.43	261.71	641.43
R_F	674.86	269.43	261.71	641.43

Supplementary Table 5. Comparison of OER activity of TS-Ir/MnO_2 electrocatalyst with other recently reported catalysts in acid solution. [12-20]

Catalyst	Electrolyte	Support	η At 10 mA cm^{-2}	η At 250 mA cm^{-2}	Mass activity (A g_{metal}^{-1})	Ref.
TS-Ir/MnO_2	0.1 M $HClO_4$	Carbon cloth	198	356	1025 at 198 mV<br>7816 at 260 mV	This work
Ir-MnO_2	0.1 M $HClO_4$	Carbon cloth	275	502	54 at 198 mV<br>2366 at 260 mV	This work
$Ir_{0.06}Co_{2.94}O_4$	0.1 M $HClO_4$	Au electrode	292	--	2511 at 300 mV	[12]
SS Pt-RuO_2 HNSs	0.5 M H_2SO_4	GCE	228	282 (100 mA cm^{-2})	--	[13]
Ru_1-Pt_3Cu	0.1 M $HClO_4$	RDE (GC)	280	--	779 at 250 mV	[14]
$IrO_x/SrIrO_3$	0.5 M H_2SO_4	$SrTiO_3$	270-290	--	--	[15]
IrO_X/9r-$BaIrO_3$	0.5 M H_2SO_4	GCE	230	--	168 at 230 mV	[16]
$Ru_1Ir_1O_x$	0.5 M H_2SO_4	Ti-plates	204	300* (120 mA cm^{-2})	1124 at 300 mV	[17]
GB-$Ta_{0.1}Tm_{0.1}$ $Ir_{0.8}O_{2-\delta}$	0.5 M H_2SO_4	RDE (Au)	198	260* (100 mA cm^{-2})	3126 at 266 mV	[5]
$Mn_{0.73}Ru_{0.27}$ $O_{2-\delta}$	0.5 M H_2SO_4	GCE	208	--	879 at 270 mV	[18]
12Ru/MnO_2	0.1 M $HClO_4$	GCE	161	--	1264 at 165 mV	[7]
$NaRuO_2$	0.1 M $HClO_4$	Ti-plates	225	--	42 at 250 mV	[19]
Li-IrO_X	0.5 M H_2SO_4	RDE	270	--	100 at 290 mV	[20]

*Data are not specifically reported in the article, obtained from the LSV curves.
Note: GCE, glassy carbon electrode; DRE, rotating disk electrode

Supplementary Table 6. The apparent bond length of Ir–O in TS-Ir/MnO_2 under different working conditions.

Sample	Apparent bond length (Ir–O)
ex situ	1.54 Å
1.15 V	1.59 Å
1.30 V	1.59 Å
1.45 V	1.59 Å

Supplementary Table 7. Structural parameters for TS-Ir/MnO_2 electrocatalyst under different potentials extracted from quantitative EXAFS curve-fitting using the ARTEMIS module of IFEFFIT.

Sample	Path	N	R (Å)	$\sigma^2(10^{-3}Å^2)$	ΔE_0(eV)	R-factor
ex situ	Ir-O	4.3±0.3	1.94±0.01	5.6±0.5	9.9±1.5	0.004
1.15V	Ir-O	4.3±0.2	1.96±0.01	5.9±0.5	9.7	0.006
	Ir-O_2	1.1±0.2	2.08±0.01	3.6±0.3		
1.30V	Ir-O_1	4.2±0.2	1.96	5.5±0.8	9.7	0.003
	Ir-O_2	1.3±0.2	2.08±0.01	3.2±0.2		
1.43V	Ir-O_1	3.3±0.2	1.96	5.2±1.2	9.7	0.005
	Ir-O_2	1.2±0.2	2.07±0.01	3.4±0.8		

N, coordination number; R, bond length; σ^2 Debye-Waller factor; ΔE_0 inner potential shift

Reference

1 Xu, Z. *et al.* Light-driven orderly assembly of Ir-atomic chains to integrate a dynamic reaction pathway for acidic oxygen evolution. *Angew. Chem. Int. Ed.* **62**, e202301128 (2023).

2 Kakati, U. *et al.* Iridium incorporation into MnO_2 for an enhanced electrocatalytic oxygen evolution reaction. *ChemCatChem* **15**, e202201549 (2023).

3 Ghadge, S. D. *et al.* Experimental and theoretical validation of high efficiency and robust electrocatalytic response of one-dimensional (1D)(Mn, Ir) O_2: 10F nanorods for the oxygen evolution reaction in PEM-based water electrolysis. *ACS Cata.* **9**, 2134-2157 (2019).

4 Lee, C. *et al.* Catalyst-support interactions in Zr_2ON_2-Supported IrO_x electrocatalysts to break the trade-off relationship between the activity and stability in the acidic oxygen evolution reaction. *Adv. Fun. Mater.* **33**, 2301557 (2023).

5 Hao, S. *et al.* Torsion strained iridium oxide for efficient acidic water oxidation in proton exchange membrane electrolyzers. *Nat. Nanotechno.* **16**, 1371-1377 (2021).

6 Chen, J. *et al.* Low-coordinate iridium oxide confined on graphitic carbon nitride for highly efficient oxygen evolution. *Angew. Chem. Int. Ed.* **58**, 12540-12544 (2019).

7 Lin, C. *et al.* In-situ reconstructed Ru atom array on α-MnO_2 with enhanced performance for acidic water oxidation. *Nat. Catal.* **4**, 1012-1023 (2021).

8 Wu, Z. *et al.* Hexagonal defect-rich MnOx/RuO_2 with abundant heterointerface to modulate electronic structure for acidic oxygen evolution reaction. *Adv. Fun. Mater.* 2307010.

9 Qin, Y. *et al.* RuO_2 electronic structure and lattice strain dual engineering for enhanced acidic oxygen evolution reaction performance. *Nat. Commun.* **13**, 3784 (2022).

10 Zhang, X., Feng, C., Dong, B., Liu, C. & Chai, Y. High-voltage-enabled stable cobalt species deposition on MnO_2 for water oxidation in acid. *Adv. Mater.* e2207066 (2023).

11 Wang, Z. *et al.* Influence of the MnO_2 phase on oxygen evolution reaction performance for low-loading iridium electrocatalysts. *ChemElectroChem* **8**, 418-424 (2021).

12 Shan, J. *et al.* Short-range ordered iridium single atoms integrated into cobalt oxide spinel structure for highly efficient electrocatalytic water oxidation. *J. Am. Chem. Soc.* **143**, 5201-5211 (2021).

13 Wang, J. *et al.* Single-site Pt-doped RuO_2 hollow nanospheres with interstitial C for high-performance acidic overall water splitting. *Sci. Adv.* **8**, eabl9271 (2022).

14 Yao, Y. *et al.* Engineering the electronic structure of single atom Ru sites via compressive strain boosts acidic water oxidation electrocatalysis. *Nat. Catal.* **2**, 304-313 (2019).

15 Seitz, L. C. *et al.* A highly active and stable IrOx/$SrIrO_3$ catalyst for the oxygen evolution reaction. *Science* **353**, 1011-1014 (2016).

16 Li, N. *et al.* Identification of the active-layer structures for acidic oxygen evolution from 9R-BaIrO3 electrocatalyst with enhanced iridium mass activity. *J. Am. Chem. Soc.* **143**, 18001-18009 (2021).

17 He, J., Zhou, X., Xu, P. & Sun, J. Regulating electron redistribution of intermetallic iridium oxide by incorporating Ru for efficient acidic water oxidation. *Adv. Energy*

Mater. **11**, 2102883 (2021).
18 Wang, K. *et al.* Highly active ruthenium sites stabilized by modulating electron-feeding for sustainable acidic oxygen-evolution electrocatalysis. *Energy Environ. Sci.* **15**, 2356-2365 (2022).
19 Laha, S. *et al.* Ruthenium oxide nanosheets for enhanced oxygen evolution catalysis in acidic medium. *Adv. Energy Mater.* **9**, 1803795 (2019).
20 Gao, J. *et al.* Breaking long-range order in iridium oxide by alkali ion for efficient water oxidation. *J. Am. Chem. Soc.* **141**, 3014-3023 (2019).
